# An objective method for the production of isopach maps and implications for the estimation of tephra deposit volumes and their uncertainties

**DOI:** 10.1007/s00445-015-0942-y

**Published:** 2015-06-17

**Authors:** S. L. Engwell, W. P. Aspinall, R. S. J. Sparks

**Affiliations:** School of Earth Science, University of Bristol, Queens Road, Bristol, BS8 1RJ UK; Istituto Nazionale di Geofisica e Vulcanologia, Sezione di Pisa, Via della Faggiola 32, 56126 Pisa, Italy

**Keywords:** Tephra volume, Isopach maps, Uncertainties, Cubic B-spline

## Abstract

Characterization of explosive volcanic eruptive processes from interpretation of deposits is a key for assessing volcanic hazard and risk, particularly for infrequent large explosive eruptions and those whose deposits are transient in the geological record. While eruption size—determined by measurement and interpretation of tephra fall deposits—is of particular importance, uncertainties for such measurements and volume estimates are rarely presented. Here, tephra volume estimates are derived from isopach maps produced by modeling raw thickness data as cubic B-spline curves under tension. Isopachs are objectively determined in relation to original data and enable limitations in volume estimates from published maps to be investigated. The eruption volumes derived using spline isopachs differ from selected published estimates by 15–40 %, reflecting uncertainties in the volume estimation process. The formalized analysis enables identification of sources of uncertainty; eruptive volume uncertainties (>30 %) are much greater than thickness measurement uncertainties (~10 %). The number of measurements is a key factor in volume estimate uncertainty, regardless of method utilized for isopach production. Deposits processed using the cubic B-spline method are well described by 60 measurements distributed across each deposit; however, this figure is deposit and distribution dependent, increasing for geometrically complex deposits, such as those exhibiting bilobate dispersion.

**Electronic supplementary material:**

The online version of this article (doi:10.1007/s00445-015-0942-y) contains supplementary material, which is available to authorized users.

## Introduction

Quantification of eruption volumes and estimation of associated uncertainties is a fundamental task in volcanology and is a key to characterizing eruption magnitudes (M, where M = log10(m)—7.0; m = erupted mass in kg) and their relationship with eruption frequency (e.g., Mason et al. 2004). A number of methods have been proposed to estimate eruptive volumes, for example, comparison of near vent deposits with those from other eruptions where volume is well constrained (Bond and Sparks 1976), analysis of components within deposits (Walker 1973), regression analysis of thickness data (Burden et al. 2013), and application of tephra dispersion models for inversion of mass per unit area data (Connor and Connor 2006). Analysis of isopach maps using semi-empirical descriptive models (e.g., Tsuya 1955; Thorarinsson 1954; Walker 1980; Pyle 1989, 1995; Fierstein and Nathenson 1992; Bonadonna and Houghton 2005; Sulpizio 2005; Bonadonna and Costa 2012) remains the most widely applied method, however, and toolboxes have been produced in which these different methods are applied simultaneously to isopach data to provide a range of eruptive volume estimates (Daggitt et al. 2014) and their uncertainties (Biasse et al. 2014). In addition to volume estimation, isopach maps are crucial for identifying tephra dispersal patterns and hence dominant wind direction(s).

The accuracy of isopach map derived volume estimates depends on quality and quantity of thickness data and how isopach contours are drawn. This means that assessment of tephra fall deposit volumes is limited, intrinsically, by uncertainties in field data (Bonadonna and Costa 2012; Engwell et al. 2013). Volume estimates often require extrapolation beyond the areas for which there are data, and here, the choice of fitting function (e.g., exponential, power law, or Weibull) is crucial (Klawonn et al. 2014a). Volume estimates for the same deposit may differ by 50 % or more when different volume calculation methods are used or different assumptions are applied (Sulpizio 2005). Studies on well-documented proximal deposits have highlighted the importance of depositional processes, particularly the effect of particle Reynolds number (e.g., Bonadonna et al. 1998) and aggregation (e.g., Carey and Sigurdsson 1982; Folch et al. 2010) on deposit thinning trends and because controlling processes can vary with distance from source, thinning rates may be complex. While depositional processes in proximal to medial reaches are fairly well defined, processes in distal reaches are less well understood and in these locations thinning trends are often difficult to determine because of deposit erosion or redeposition.

There is no standardized method for preparing isopach maps, which are generally hand drawn. While the process is objectively based, it is also personalized: Klawonn et al. (2014b) showed that a number of geologists presented with the same thickness measurements can produce very different maps. The quality of a hand-drawn isopach contour reflects a number of tangible and some less tangible factors, including number of thickness measurements, their spatial distribution, errors in field measurements, the local knowledge and experience of the geologist drawing the isopach contours, and underlying assumptions regarding processes of tephra deposition and therefore isopach shape. Some of these influences are difficult to retrieve from published maps or even from the data themselves. In fact, when drawing isopachs, scientists are likely to weight measurements mentally according to their field characteristics, but such conditioning assumptions are typically not recorded.

An implicitly more objective option for drawing isopach maps is to fit thickness data to a surface described mathematically using formal interpolation methods. Such interpolation methods have previously been used to infer rainfall patterns in areas where there are few data (e.g., Hofierka et al. 2002) and in topographical analysis (Mitasova and Hofierka 1993). Rhoades et al. (2002) applied a local regression model to the Taupo Plinian Pumice fall deposit using the statistical method of Chambers and Hastie (1992). In this case, a weighted average of neighboring data points was used and apparent outliers given weights such that their effect on the resulting isopach map is reduced. While Bursik and Sieh (2013) and Bisson and Del Carlo (2013) both apply mathematical models, their methods are limited to the use within GIS frameworks and do not incorporate estimates of thickness measurement uncertainty or provide estimates of uncertainty for derived volumes.

Here, we present a method for producing isopach contours using cubic B-splines under tension. The cubic B-spline method, a spatial statistical method, reflects the geometry of the deposit and spacing of measurements, and does not take into account the physics of processes that occur during eruption or sedimentation. While inversion of tephra dispersion and deposition models can be used to estimate eruptive volumes, current models cannot represent all deposition processes accurately, and therefore, other methods, such as that described here, are required to characterize volumes of tephra deposits objectively.

The method is applied to a range of examples, with different measurement spatial resolution, eruption style and magnitude, and dispersion conditions, e.g., low to high wind conditions. The Fogo member A deposit (Walker and Croasdale 1971) is used to demonstrate the method. The resulting isopach map is compared with the original isopach map of Walker and Croasdale (1971), and volume is estimated by direct integration of the cubic B-spline surface and assuming exponential thickness decay (Pyle 1989; Fierstein and Nathenson 1992). Measurements are weighted such that associated errors are included, and their impact on the spline-generated isopachs is analysed. The method is also applied to deposits from a number of other volcanoes (Askja, Taupo, El Chichon, Cerro Negro, and Mount St. Helens) and to subsets of thickness data to assess variance in calculated volume related to the number of measurements.

## Method

We apply a mathematical model to interpret and interpolate tephra thickness data. Spline interpolation is a methodology whereby the interpolant is a piecewise polynomial, i.e., the function is defined by multiple sub-functions that each apply to a certain interval of the domain. In the 2-D case of cubic B-spline interpolation, the solution can be envisaged in terms of deformation of a thin elastic ‘plate’ under tension (Inoue 1986). The surface of this notional plate is locally pulled towards each data point by the numerical equivalent of a spring whose individual stiffness is inversely proportional to data variance (Inoue 1986). Here, we fit cubic B-splines to tephra thickness data using the FORTRAN code of Inoue (1986).

The cubic B-splines method of Inoue (1986) is based on minimization of a weighted sum of the least squares spline fit to the data points (*d*_*p*_)(*p = 1 ….. n*) and the first and second derivatives. The first derivative is associated with the spline tension and serves to minimize fluctuations at the spline boundaries, while the second derivative is related to the roughness of the spline. The aim is to find a continuous distribution approximation ϕ, which minimizes the approximation error. To this end, the smoothing fit to the data is determined by the least squares norm ⊓ composed of data residuals, the first and second derivatives:

1 $$ \sqcap =\parallel {R}^2+\parallel {J}^2={\displaystyle \sum_{p=1}^n}{W}_{\mathrm{p}}{\left({\phi}_{\mathrm{P}}-{d}_{\mathrm{p}}\right)}^2+\frac{1}{l_{\mathrm{u}}^2}{\displaystyle \underset{\Omega}{\iint }}\left[{W}_1\left({\phi}_x^2+{\phi}_y^2\right)+{W}_2\left({\phi}_{xx}^2+2{\phi}_{xy}^2 + {\phi}_{yy}^2\right)\right]\ dx\  dy $$

where *R* refers to the misfit of the function to the data, *J* represents the roughness, ϕ is the smoothing function, Ω is the domain, *W* represent weightings, *l*_u_ is a unit length, and subscripts *x*, *y*, etc. express differentiations. The optimum smoothing function $\widehat{\phi}$ through the thickness measurements is defined over the area of interest (Ω*x* × Ω*y*) in the *x-y* plane, where *x* refers to the longitude and *y* the latitude of the area in question. The smoothing function in tensor product form for the cubic B-spline basis functions *Fi(x)* and *Gj(y)* is described as:

2 $$ \widehat{\phi}\left(x,y\right) = {\displaystyle \sum_{i=1}^{M_x+3}}{\displaystyle \sum_{j=1}^{M_y+3}}{c}_{\mathrm{i}\mathrm{j}}{F}_{\mathrm{i}}(x){G}_{\mathrm{j}}(y) $$

where *Mx* and *My* are the number of coefficients in the *x* and *y* domains, respectively, and *c*_ij_ are the unknown coefficients. The cubic B-spline bases have equally-spaced knots, i.e., divisions of the area in question, *ξ*_− 3_ … *ξ*_0_, *ξ*_− 1_ … *ξ*_M_, *ξ*_M_ … *ξ*_M + 3_ and, as an example, the nonzero parts of the basis *F*_i_*(x)*, can be written as:

3 $$ {F}_{\mathrm{i}}(x)=\left\{\begin{array}{c}\hfill \begin{array}{cc}\hfill 0\hfill & \hfill \left(x<{\xi}_{i-4}\right)\hfill \\ {}\hfill {B}_1\left[\left(x-{\xi}_{i-4}\right)/f\right]\hfill & \hfill \left({\xi}_{i-4}<x<{\xi}_{i-3}\right)\hfill \end{array}\hfill \\ {}\hfill \begin{array}{cc}\hfill {B}_2\left[\left(x-{\xi}_{i-3}\right)/f\right]\hfill & \hfill \left({\xi}_{i-3}<x<{\xi}_{i-2}\right)\hfill \\ {}\hfill {B}_3\left[\left(x-{\xi}_{i-2}\right)/f\right]\hfill & \hfill \left({\xi}_{i-2}<x<{\xi}_{i-1}\right)\hfill \end{array}\hfill \\ {}\hfill \begin{array}{cc}\hfill {B}_4\left[\left(x-{\xi}_{i-1}\right)/f\right]\hfill & \hfill \left({\xi}_{i-1}<x<{\xi}_i\right)\hfill \\ {}\hfill 0\hfill & \hfill \left({\xi}_i\le x\right)\hfill \end{array}\hfill \end{array}\ \right. $$

where *f* is the knot interval and *B*_i_*(r)* are the cubic polynomials:

4 $$ \begin{array}{c}\hfill {B}_1(r)=\frac{r^3}{6}\hfill \\ {}\hfill {B}_2(r)=\frac{-3{r}^3+3{r}^2+3r+1}{6}\hfill \\ {}\hfill {B}_3(r)=\frac{3{r}^3-6{r}^2+4}{6}\hfill \\ {}\hfill {B}_4(r)=\frac{-{r}^3+3{r}^2-3r+1}{6}\hfill \end{array} $$

By substituting Eq. 2 into Eq. 1, and assuming ∂⊓/∂*c*_*j*_ = 0 for j = 1, …, M, i.e., ⊓ is minimized, M linear coefficients for the coefficients *c* can be written in matrix form whereby:

5 $$ \mathbf{K}c=\mathbf{b} $$

The coefficient matrix **K** is decomposed as:

6 $$ \boldsymbol{K}={\boldsymbol{K}}^{\boldsymbol{d}}+{\boldsymbol{K}}^{10}+{\boldsymbol{K}}^{01}+{\boldsymbol{K}}^{20}+2{\boldsymbol{K}}^{11}+{\boldsymbol{K}}^{02} $$

where

7 $$ {K^{\mathrm{d}}}_{{\mathrm{i}\mathrm{ji}}^{\prime }{\mathrm{j}}^{\prime }}={\displaystyle \sum_{p=1}^n}{w}_{\mathrm{p}}{F}_{\mathrm{i}}^{\mathrm{p}}{G}_{\mathrm{i}}^{\mathrm{p}}{F}_{{\mathrm{i}}^{\prime}}^{\mathrm{p}}{G}_{{\mathrm{j}}^{\prime}}^{\mathrm{p}} $$

and

8 $$ {K}_{{\mathrm{i}\mathrm{j}\mathrm{i}}^{\prime }{\mathrm{j}}^{\prime}}^{\mathrm{hl}}=\frac{W_{k+1}}{l_u^2}{\displaystyle \underset{\Omega_{\mathrm{x}}}{\int }}{F}_{\mathrm{i}}^{(k)}{F}_{{\mathrm{i}}^{\prime}}^{(k)}dx{\displaystyle \underset{\Omega_{\mathrm{y}}}{\int }}{G}_{\mathrm{j}}^{(l)}{G}_{{\mathrm{i}\mathrm{j}}^{\prime}}^{(l)} dy $$

and the vector **b** is given as:

9 $$ {b}_{ij}={\displaystyle \sum_{p=1}^n}{w}_p{F}_i^p{G}_i^p{d}_p $$

Here, *w*_p_ are weights that describe uncertainty in thickness measurements *d*_p_. Further explanation and derivations are provided in Inoue (1986).

The method requires four fitting parameters; tension (τ), roughness (ρ), number of divisions of the area (i.e., spline knot spacing), and measurement weights (*w*_p_). Applying the method, we try to satisfy two conflicting aims, namely producing a good fit to the data while avoiding excessive local variation in the resultant surface that becomes unphysical. Tension is added to the spline to minimize distortions of the surface between data points, such that the fitted surface remains physically meaningful and plausible for subsequent analysis. The tension value can vary between 0 and 1. When no tension is applied to the data (τ = 0), unphysical bumps characterized by large thickness variations can appear in areas far from constraining data points. However, applying high tension (τ = 1) can cause sharp, unrealistic angles in the surface at data points (Appendix 1). Following previous studies (e.g., Inoue 1986; Bauer et al. 1998), τ is set to 0.99 to prevent anomalous highs forming in extrapolated areas with little or no data.

The knot spacing is determined based on the deposit extent and number of measurements. For well-documented deposits, more knots can be used to show local variation in thickness trends. When data are poorly spaced across the deposit extent, fewer knots are used, so a smoother surface is produced reflecting global rather than local thinning trends (see Appendix 2 for further details). In the examples presented here, a knot spacing of 10 km is used.

In the first instance, weights of one were applied (*w*_p_ = 1), so that all data points had equal weighting. Varying the weighting of all thickness measurements by the same amount results in a volume estimate that varies by the same factor. However, an advantage of the spline method is that each measurement may be weighted individually, for example, according to measured thickness uncertainty or according to proportional error. But, in general, values of uncertainty are not presented for thickness measurements reported in the volcanological literature. However, uncertainty estimates are available for the Fogo Member A example (Engwell et al. 2013) and here, in addition to being weighted equally, the Fogo A dataset is also weighted according to these uncertainties, as described below.

**Fig. 1:**
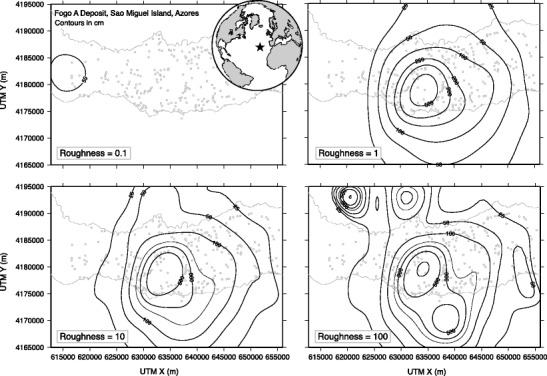
Fogo Member A deposit on Sao Miguel, Azores (location denoted by star on inset map): effect of changing spline roughness parameter on modelled isopach mapping. A very small roughness parameter (first panel) generates a spatially-flat function resulting in an unrealistically smooth deposit thickness surface, with known local trends obscured. High roughness values produce complex surfaces that do not reproduce overall trends in deposit thickness (fourth panel). Intermediate roughness values (second and third panels) produce plausible alternative iospach maps

Roughness (ρ) controls the balance between fit to data and overall model surface roughness. Roughness values that are too small give large residuals relative to observational errors on individual measurements. However, large roughness values give too much weight to measurements with no consideration to global trends in data (Inoue 1986). Typical values used for ρ lie between 0.1 and 1000, where 0.1 gives a very smooth fit (essentially a linear best fit plane through the data) while a roughness of 1000 produces exaggerated variations in the surface (Fig. 1). Small values of roughness are sufficient where there is a little local variation in data. Higher roughness values are required to reflect large variations in data, but too high values of roughness obscure the global trend. Therefore, choice of roughness value is guided to some extent by the visual credibility of the resulting fitted thickness trend surface. In the method, we determine the most appropriate value by using roughness values that range over six orders of magnitude (0.01, 0.1, 1, 10, 100, and 1000) and assess the resulting map based on fit to data points and visual credibility. Surfaces that are too flat and therefore do not accurately represent global changes in data, and those that are too rough with overly complicated contour patterns are discarded. Thus, there is some element of subjective judgment in parameter choice with a spline-derived pattern that has—through adjustment of the roughness parameter—a broad similarity to the decision process for visually-identified, hand-drawn isopach maps. The spline method provides results that produce the lowest objective data-fitting error, either in terms of global trends in the data or for highlighting very local variations in deposit thickness, depending on the choice of spline parameters.

Fitted results are in the form of a gridded dataset of interpolated thickness values across the specified x-y domain. This dataset is processed using GMT software (Wessel and Smith 1991), where the function *grdcontour* is used to contour results at user specified thickness, and *grdvolume* is used to determine area within each contour to enable the production of log thickness versus square root isopach area plots. The volume between each isopach contour and the interpolated surface was quantified by direct integration of the spline-derived surface. Volumes were derived from the cubic B-spline isopach maps assuming exponential thickness decay following Pyle (1989) and Fierstein and Nathenson (1992). These methods do not enable extrapolation of the thickness trends to areas where there is no deposit exposure, and therefore estimates constitute a minimum erupted volume, thus allowing direct comparison with volumes determined by integration of the spline surface. Consideration of uncertainties associated with choice of thickness decay assumption (e.g., exponential (Pyle 1989), power law (Bonadonna and Houghton 2005), or Weibull (Bonadonna and Costa 2012)) is discussed in detail in Klawonn et al. (2014a, b) and is not pursued further here.

The method was applied to a number of examples representing a range in eruptive styles and magnitude (Table 1). The Fogo member A deposit was produced during a trachytic Plinian eruption approximately 5000 years ago (Moore 1990). Walker and Croasdale (1971) and Bursik et al. (1992) separated the deposit into two volumetrically dominant fallout deposits, a lower syenite-poor Plinian fall and an overlying, syenite-rich Plinian fall deposit. The lower syenite-poor Plinian deposit formed while there was a southerly wind, while the coarser grained syenite-rich deposit has a near-axisymmetric distribution and an inferred Plinian plume height of 21 km (Bursik et al. 1992). Walker and Croasdale (1971) present measurements of the combined thickness of the two Plinian deposits and inferred a bulk deposit volume of 1.2 km^3^. The Walker and Croasdale (1971) Fogo member A dataset is large, with 250 measurements, distributed axisymmetrically around the vent, and as such the dataset represents one of the best in the world with regards to spatial density of measurements. However, the distribution of measurements is limited to Sao Miguel Island, and therefore only the very proximal to medial deposits are represented, with the furthermost measurement ~20 km from source. A number of measurements of ‘zero’ thickness were reported by Walker and Croasdale (1971), indicating eastern and western extents of the deposit. The deposit is poorly constrained to the north and south, where deposition occurred over the sea. This said, because dispersion is inferred to be near uniaxial, thickness trends are well represented by measurements on land. The study of Engwell et al. (2013), quantified uncertainties for the Fogo member A deposit, and therefore it was possible to weight measurements directly to uncertainties measured in the field. Engwell et al. (2013) showed that for the Fogo A deposit, the uncertainty, *y* (%), in thickness, *x* (cm), follows a power-law:

10 $$ y=29{x}^{-0.3} $$

**Table 1:** Fogo member A isopach map of Walker and Croasdale (1971): Comparison of differences between measured thicknesses and spline interpolation of published isopachs; mean and standard deviation of percentage differences for distributions shown in Fig. 2 (first row)

	Mean %	Standard deviation of %
Measured thickness vs. thickness from isopach map interpolation difference	64	95.8
Measurement error	16.2	29.8
Exponential regression fit difference	90.4	143.8
Power law regression fit difference	83	128.1

Other rows: calculated measurement error and percentage differences between exponential and power law regression fits to thickness with distance (from Engwell et al. 2013)

Therefore, interpolation was conducted by weighting data points according to this relation, with thinner distal deposits given less weight than thicker proximal deposits.

The Askja 1875 Layer D deposit is the main product of a 6.5 h-long rhyolitic Plinian eruption (Sparks et al. 1981). The deposit is coarse grained and well sorted, and proximally can be separated into five subunits (Carey et al. 2010). The medial–distal deposit is comprised of subunits D1, D3, and D5 (Carey et al. 2010) and covers an area of 7500 km^2^ (Sparks et al. 1981). Published isopachs of the deposit are elongate to the east, showing dispersal was strongly wind controlled. Thickness values were digitized from Sparks et al. (1981). The 136 measurements are well distributed in proximal areas and in distal reaches are focused in bands near perpendicular to the crosswind axis.

Thickness data for the rhyolitic 1.8 ka Taupo Plinian Pumice deposit were digitized from Walker (1981), who inferred a bulk deposit volume of 9 km^3^. Published isopachs show a strong easterly elongation, with maximum thickness displaced 20 km downwind from inferred source. In comparison to the Askja Layer D and Fogo member A eruption, there are no very proximal thickness values available, due to source location within Lake Taupo. The dataset comprises 180 measurements that are well dispersed across the medial portion of the deposit, becoming more widely spaced in distal portions.

Thickness data for the 1995 Cerro Negro (81 measurements), El Chichon 1982 Layer B (69 measurements), and Mount St. Helens May 18th 1980 (235 measurements) eruptions were obtained from the IAVCEI Commission on tephra hazard modeling (http://www.ct.ingv.it/Progetti/Iavcei/results.htm). The basaltic Cerro Negro eruption consisted of numerous small explosions over the course of 13 days and resulted in a deposit with an estimated bulk volume of 0.003 km^3^ (Hill et al. 1998). Ash was deposited up to 30 km to the west, with published isopachs showing a distinct bilobate distribution.

The El Chichon Layer B deposit resulted from a major trachyandesitic Plinian eruption with an observed column height of >17 km (Carey and Sigurdsson 1986). The resultant plume was dispersed ENE–WSW, with published isopachs showing a circular distribution. Maximum deposit thickness is found 6 km downwind from source, and the inferred bulk deposit volume is 0.79 km^3^ (Carey and Sigurdsson 1986). Thickness measurements are well distributed across the deposit extent, with spatial distribution decreasing from proximal to distal reaches.

Finally, the Mount St. Helens eruption of May 18th 1980 began as a result of a large landslide that released pressure from a shallow magma chamber resulting in a lateral blast. Large pyroclastic density currents (PDCs) followed, covering an area of 600 km^2^ (Sparks et al. 1986). From these currents, a large co-PDC plume formed, dispersing fine-grained ash to the north and east of the source (Sparks et al. 1986). Approximately 30 min after this phase, Plinian activity began (Sparks et al. 1986), with an average column height of 16 km, transporting dacitic material to the east (Sarna-Wojcicki et al. 1981; Carey and Sigurdsson 1982). The eruption lasted 9 h producing a deposit that extended 1000 km east of the source. The data used here describe both the co-PDC and Plinian deposit. While in most other examples, deposit extent is poorly constrained, in the Mount St. Helens example the downwind deposit extent is well constrained by 47 zero thickness values.

## Results

### Uncertainty in hand-drawn isopach maps

**Fig. 2:**
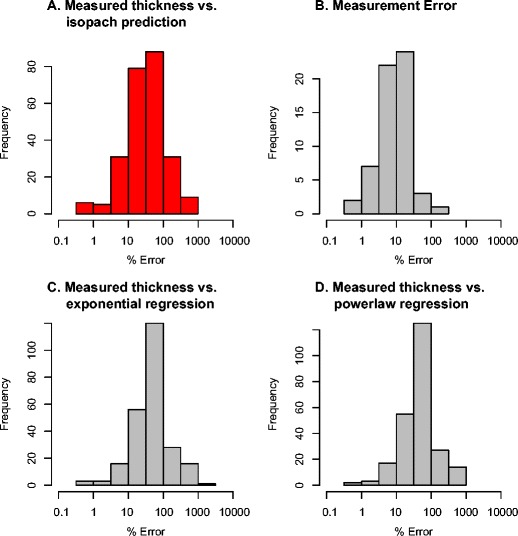
Fogo member A isopach map data from Walker and Croasdale (1971). Histograms showing differences between published measurements and thicknesses indicated by different models: **a** Spline model-based observational differences on published isopach shown in Fig. 3a; **b** human measurement errors determined using thickness data collected during field analysis of the Fogo member A deposit (Engwell et al. 2013); **c** and **d** residuals between thickness data and least squares best-fit exponential distance decay model (**c**) and power law regression distance decay (**d**)

The difference between measured thickness and thickness predicted by the hand-drawn Fogo member A isopach map of Walker and Croasdale (1971) was quantified. The published hand-drawn isopachs were digitized such that each was described by a number of points to which spline interpolation was applied to produce a smooth surface. This interpolated surface was sampled to determine predicted thickness at each measurement location. The residual between the measured thickness and the thickness predicted by the published isopach map was calculated as a percentage difference (Fig. 2a). The errors can be approximately characterised as log normal, but are skewed with a longer tail towards smaller percentage differences and errors.

Measured uncertainty on tephra thickness measurements of the Fogo member A deposit, taken from Engwell et al. (2013), is displayed in Fig. 2b and Table 1. In addition, histograms of the residuals between an exponential and a power-law least-squares regression fit to the Walker and Croasdale (1971) Fogo member A thickness measurements versus distance from source are also shown (Fig. 2c, d and Table 1). Mean uncertainty in the hand drawn isopach map is 64 %, significantly greater than the uncertainty associated with observational uncertainty of an individual tephra unit (16 %) within the Fogo member A deposit. This comparison of errors suggests that the departure of individual measurements from the smoothed surface of the isopach map of Walker and Croasdale (1971) is largely but not completely explained by measurement variation and natural variability related to depositional processes. The Fogo member A isopach, exponential (Fig. 2c) and power law (Fig. 2d) fit percentage difference distributions are similar but with different mean percentage differences of 64, 90, and 84 %, respectively. The exponential and power-law fit residuals, describing variation of measurements from expected trends with distance from source, have significantly higher standard deviation (Table 1) compared to hand-drawn isopach maps.

**Fig. 3:**
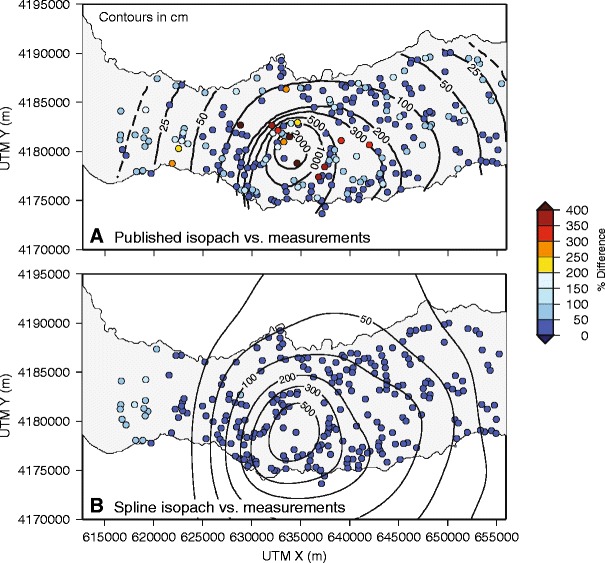
**a** Fogo member A isopach contours produced by Walker and Croasdale (1971); *markers* show data point locations, with marker colors reflecting percentage difference between thickness derived by spline interpolation of the Walker and Croasdale (1971) contours and measured thickness (see text and Table 1). **b** Percentage difference between the cubic B-spline isopach map (this paper) and the original published thickness measurements

Figure 3 displays no trend in spatial variation of difference between isopach and measured thickness of the Fogo member A deposit, with clusters of large percentage difference both proximal and distal to vent. The clustering likely reflects deposit natural variance related to depositional processes, for example, deposition at the base of a slope, but may also be attributed to systematic uncertainty resulting from the isopach map preparation process. Where a large number of measurements are available, the difference between measured thickness and the surface predicted by the hand-drawn isopach is smaller, indicating a negative correlation between measurement density and isopach uncertainty; this confirms the usual presumption that this is so.

### Cubic B-spline isopachs

The cubic-B spline method was applied to thickness data from the six case deposits. Input best-fit parameters (Table 2) were determined for each example using the method detailed above.

**Table 2:** Eruption deposits, data sources, numbers of measurements, and parameters used in cubic B-spline case studies

Deposit	Data source	Number of measurements	Roughness	Number of divisions (X by Y)
Fogo member A	Walker and Croasdale (1971)	250	1	7 × 4
Fogo 1563	Walker and Croasdale (1971)	229	1	6 × 3
Askja 1875 Layer D	Sparks et al. (1981)	136	100	17 × 11
Taupo Plinian Pumice	Walker (1981)	180	10	5 × 3
Cerro Negro 1995	Hill et al. (1998)^a^	81	100	3 × 2
El Chichon Layer B	Sigurdsson et al. (1984); Varekamp et al. (1984)^a^	69	10	5 × 4
Mount St Helens May 18th 1980	Sarna-Wojcicki et al. (1981)^a^	235	10	10 × 5

^a^ http://dbstr.ct.ingv.it/iavcei/results.htm

#### Fogo member A

**Fig. 4:**
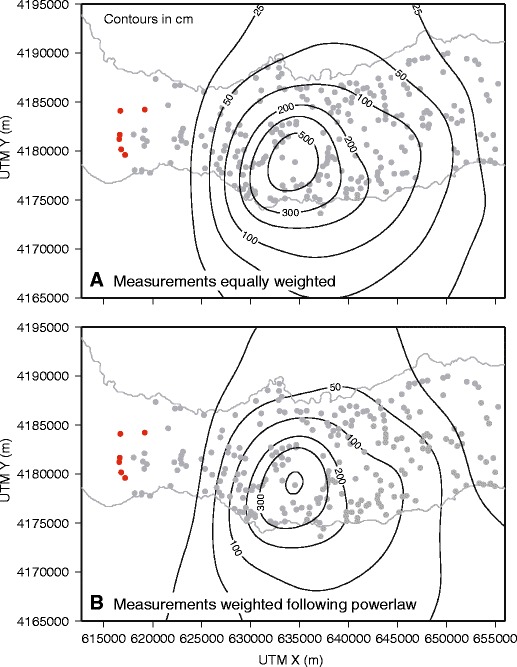
Isopach maps produced by applying a cubic B-spline model to the Fogo member A dataset (contours in cm; *red markers* represent locations where ash not observed). **a** Contours produced when all data points are equally weighted. **b** Contours obtained when data points are weighted according to measurement uncertainty (see text for details)

Isopachs from the cubic B-splines approach (Fig. 4) based on the Walker and Croasdale (1971) dataset show greater complexity than the original isopachs (Fig. 3), particularly the 100 and 200 cm isopachs, indicating non-uniaxial thickness decay with distance from source. The general shapes of the spline-derived thickness contours are similar to those in the hand-drawn contour map. However, isopachs to the west are systematically closer to the source than those to the east, indicating a greater influence of a westerly wind than originally thought (Walker and Croasdale 1971), and supporting the interpretation of Bursik et al. (1992). The results show isopach closure is dependent on spatial distribution of measurements and thickness decay trends. In the Fogo member A example, the 500, 300, 200, and 100 cm contours are closed, as they are constrained by a large number of adjacent data points and therefore spatial trends for these particular thicknesses are well defined. In comparison, the 25 cm contour cannot be reliably closed because of insufficient data to the north and south to guide the interpolated surface.

Uncertainties in tephra thickness measurements are related to syn- and post-depositional processes and measurement bias (Engwell et al. 2013). In the case of Fogo A, they vary spatially and are associated with deposit thickness. To assess the impact of uncertainty, each measurement of the Fogo member A dataset was weighted using Eq. 10. The result (Fig. 4b) is similar to that when no weighting is applied (Fig. 4a), but the 50 and 25 cm contours are less well constrained.

#### Other examples

**Fig. 5:**
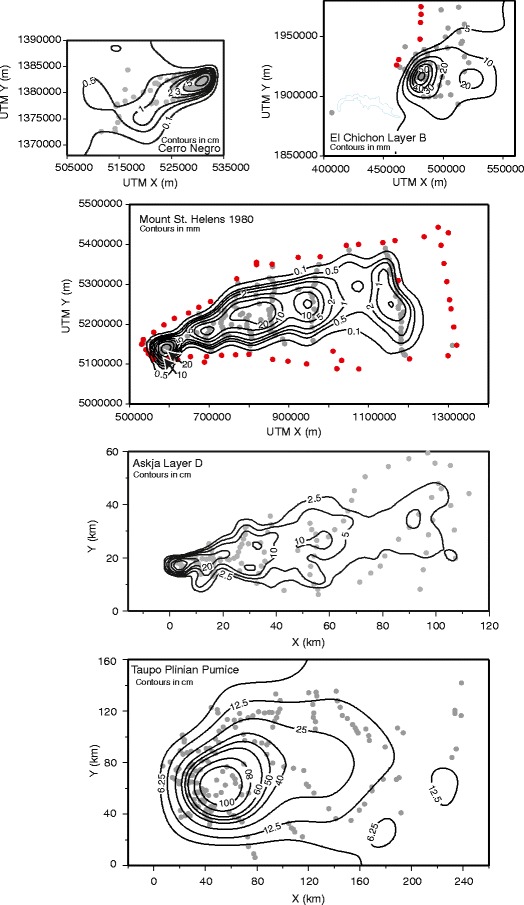
Isopach maps produced by applying a cubic-B spline model to thickness data from: Cerro Negro 1995 (Hill et al. 1998); El Chichon 1982 Layer B (Carey and Sigurdsson 1986); Mount St. Helens May 18th 1980 (Sarna-Wojcicki et al. 1981); Askja 1875 Layer D (Sparks et al. 1981); and Taupo Plinian Pumice (Walker 1981). *Red* markers denote reported locations where no ash was observed

**Fig. 6:**
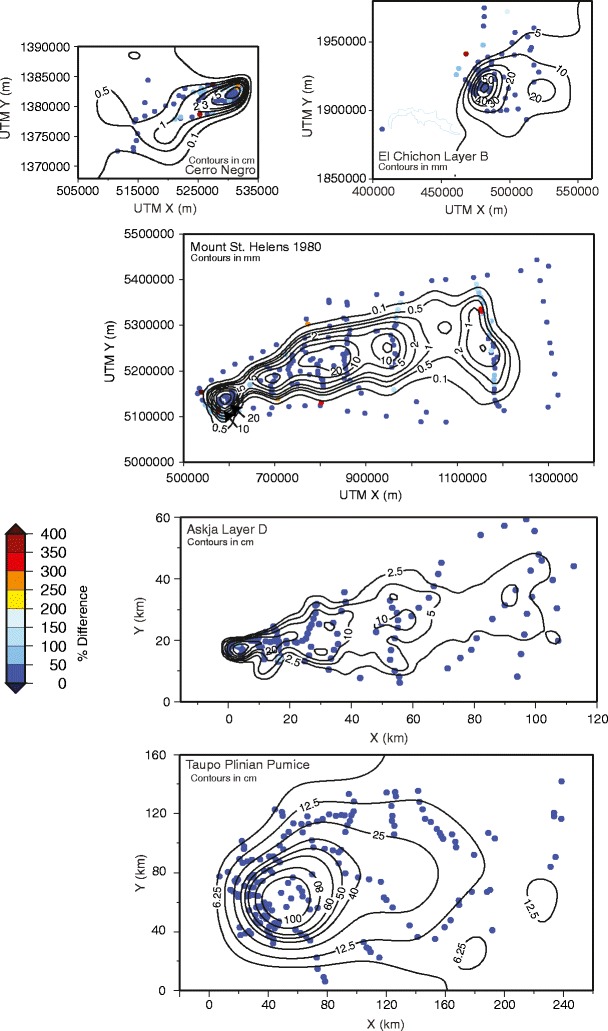
Percentage error between cubic B-spline derived isopachs and thickness measurements for each of the isopach maps in Fig. 5

Isopach maps were also produced for the Askja Layer D, Taupo Plinian Pumice, Mount St Helens, El Chichon Layer B, and Cerro Negro deposits. The resulting maps (Fig. 5) are more complex than the published hand-drawn examples. The general isopach patterns, however, are broadly similar in that the main dispersal axis is clearly identified. In each example, locations can be identified where there are insufficient data to generate closed isopach contours, for example, the 12.5 and 6.25 cm contours in the Taupo Plinian Pumice example (Fig. 5). In the examples, it is not possible to define the full extent of the deposit (i.e., where thickness goes to zero), because, with the exception of the Fogo member A and Mount St. Helens examples, the datasets do not contain information identifying locations where tephra is absent. Plotting percentage difference between spline derived isopachs and measurements (Figs. 3b and 6) shows differences are relatively consistent across the extent of each deposit.

### Thinning trends and volumes estimates

**Fig. 7:**
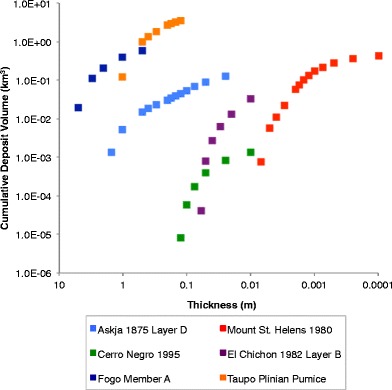
Tephra volume (km^3^) determined from integration of the cubic B-spline derived thickness surfaces for the six deposits on Fig. 5. Each point represents the volume estimate for a mapped deposit above a given thickness contour (m)

Volumes were estimated by direct integration of the spline-derived surface (Table 3, Fig. 7). Because the volume only takes into account the deposit contained within closed isopachs (i.e., there is no extrapolation beyond closed isopachs and therefore results constitute a minimum volume), it is not possible to compare results directly with those published previously. Volumes are less than, but of the same order of magnitude, as those predicted using the exponential decay integration methods of Pyle (1989) and Fierstein and Nathenson (1992) (Table 3). In each example, cumulative volume versus log thickness can be described by an exponential trend (Fig. 7), with greatest volume contained in proximal deposits, while the most distal deposits contribute a comparatively small volume.

**Table 3:** Calculated volumes and thickness decay with isopach area for eruption deposits

Deposit	Volume under surface (km^3^) (min isopach)		Tmax (m)	Thickness half distance (bt) (km)	Bulk volume (km^3^)	Volume difference spline model vs. published isopachs (%)
Fogo member A	0.57 (50 cm)	Published	32	1.87	1.5	
		Spline	9	2.96	1.0	−33
		Weighted Spline	6.3	3.31	0.9	−40
Askja 1875 Layer D	0.126 (2.5 cm)	Published	13.6	2.46		
				5.75	0.17	
		Spline	8.4	1.72		
				6.40	0.1	−40
Taupo Plinian Pumice	3.6 (12.5 cm)	Published	1.98	17.78	8.2	
		Spline	1.76	17.00	6.7	−18
Cerro Negro 1995	0.0019 (0.5 cm)	Published	1.48	0.28		
				1.20	0.003	
		Spline	0.24	0.83		
				1.84	0.0035	+14
El Chichon 1982 Layer B	0.032 (1 cm)	Published	0.08	7.24	0.06	
		Spline	0.07	10.86	0.10	+40
Mount St Helens May 18th 1980	0.43 (0.1 mm)	Published	0.87	1.75		
				32.59	1.2	
		Spline	0.11	9.78		
				23.00	0.7	−40

Second column shows volumes determined by integration of the cubic B-spline surface, with minimum isopach shown in *brackets*. Thickness decay rates for the Fogo A, Taupo Plinian Pumice and El Chichon deposits can be described by a single exponential trend and volumes were calculated following the method of Pyle (1989). Thickness decay rates of the Askja 1875, Cerro Negro and Mount St. Helens deposits are described by two exponential segments and thickness half distance for each segment is given. For these examples volume was determined using the method of Fierstein and Nathenson (1992)

**Fig. 8:**
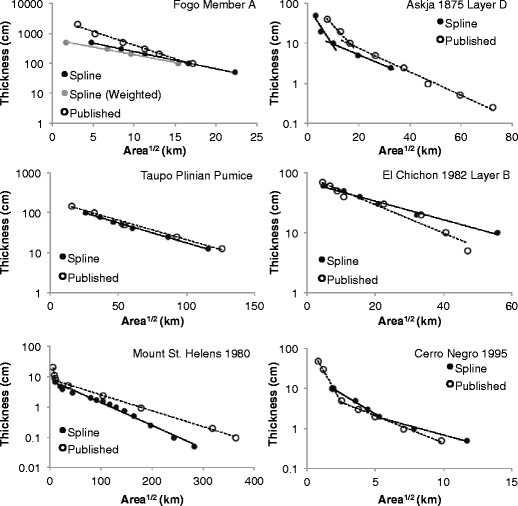
Log thickness versus square root area plots for published and cubic B-spline produced isopach maps (after Pyle 1989)

Semi-log plots of thickness versus square root area enclosed by spline-generated isopach contours were constructed, and trends are compared with hand-drawn isopachs following the method of Pyle (1989; Fig. 8). In four of the six examples, contour areas are smaller than the hand-drawn contours for the spline-generated isopachs (Fogo member A, Askja Layer D, Taupo Plinian Pumice, and Mount St. Helens). The Askja Layer D and Taupo Plinian Pumice examples show similar thinning rates between the spline- and hand-drawn isopachs. The Cerro Negro, Fogo member A, and El Chichon Layer B spline-generated isopachs all indicate much slower thinning rates with distance than those predicted by the hand-drawn isopachs, while in the Mount St. Helens example, the thinning rate is much higher than that published previously (Pyle 1989).

The Fogo member A, El Chichon Layer B, and Taupo Plinian Pumice examples are well described by a single exponent, while the Askja Layer D, Cerro Negro, and Mount St Helens examples each require two exponential segments. Thinning trends are described by modeled maximum thickness and thickness half-distance (bt; Table 3). The maximum thickness for the spline-produced isopachs is consistently less than that determined from hand-drawn isopachs. There is no systematic relationship between thickness half-distance between the hand-drawn and spline-generated isopachs.

Volumes of deposits described by simple exponential decay (Fogo A; El Chichon Layer B and Taupo Plinian Pumice) were calculated using the method of Pyle (1989). The Askja Layer D, Mount St. Helens, and Cerro Negro deposits are best described using multiple exponential segments, and the method of Fierstein and Nathenson (1992) was applied (Table 3). In the cases of Fogo member A, Askja Layer D, Taupo Plinian Pumice, and Mount St Helens, the volume estimated using spline-generated isopachs is between 20 and 40 % smaller than that determined using the hand-drawn isopachs. The volume determined for the Cerro Negro and El Chichon Layer B is greater than those determined using the hand-drawn isopach map (14 and 40 %, respectively).

### Uncertainty in isopachs associated with data set size

**Fig. 9:**
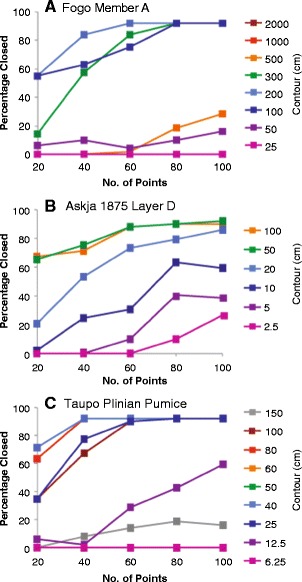
Percentages of number of closed contours obtained for different thicknesses using sub-sampled measurement datasets of different sizes for: **a** Fogo member A deposit; **b** Askja 1875 Layer D deposit; and **c** Taupo Plinian Pumice deposit. For each subset size, the sample number of data points were randomly selected from the complete dataset fifty times, interpolation was applied and a thickness contour map was produced allowing a count of number of closed contours (*colored lines* represent contour thickness in cm)

Tephra fall deposits are typically described by thickness datasets of 10 to 100 measurements. The Fogo member A, Askja Layer D, and Taupo Plinian Pumice datasets are exceptionally large, containing 250, 136, and 180 measurements, respectively. In each of these examples, the relationship between number of data points and quality of the produced isopach map and subsequent error estimates was investigated. Different numbers (10, 20…*n*…100) of data points were randomly sampled from each dataset. Each value of *n* was sampled 50 times from the total dataset and spline interpolation applied to each subset. Contour maps were produced, and the number of closed isopachs was recorded for each interpolated surface (Fig. 9). As the value of *n* data points increases, the percentage of solutions resulting in a closed contour of a particular value increases. The results indicate that at least 60 measurements widely distributed across the deposit are required for isopach contours to be consistently closed for each dataset. Further, more measurements are required if measurements are not well spatially distributed around the vent.

**Fig. 10:**
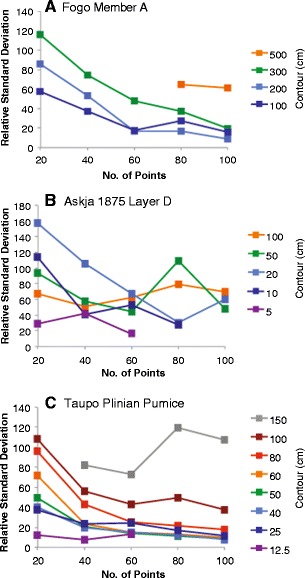
Standard deviation of area (km^2^) within each contour (each coloured line reflects a different contour value) expressed as a percentage of mean area for the **a** Fogo member A, **b** Askja 1875 Layer D, and **c** Taupo Plinian Pumice deposits

To test the relationship between area within a given contour and number of data points, the standard deviation as a percentage of the mean area within each contour for each subset size (Fig. 10) was calculated. The results follow those of number of closed contours, with percentage deviation decreasing dramatically with increased number of data points used. Once more than 60 data points are used, the reduction in standard deviation with increasing *n* is not significant. For example, in the Fogo member A 200 cm contour case, percentage of closed isopachs is 55 % when 20 measurements are used, 83 % when 40 measurements are used, and 92 % when 60 measurements are used.

### Uncertainty in eruptive volume

**Fig. 11:**
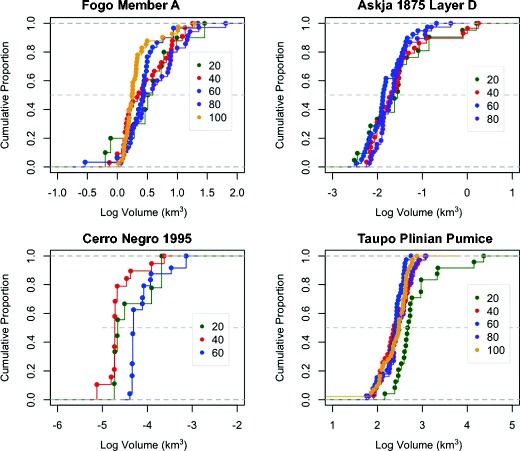
Cumulative density function of volumes determined for four deposits using spline-derived thickness contour maps produced using data subsets for each case, to which the method of Pyle (1989) is applied. The volume range and therefore uncertainty decrease with increased number of measurements, while the median volume is similar to that determined using the complete dataset for each example (see Table 4 for values and text for discussion)

To determine the effect of dataset size on volume estimates, 50 isopach maps were generated for the Fogo member A, Taupo Plinian Pumice, Askja Layer D, and Cerro Negro examples using subsets of data points (e.g., 20, 40, 60, 80, and 100). The Cerro Negro dataset contains fewer data points (<100) than the other examples, and therefore volumes were calculated using only 20, 40, and 60 measurements. A volume was calculated for each map for cases with more than three closed isopachs, and the cumulative distribution of volumes for each subset size is shown in Fig. 11. Volumes determined using a smaller number of measurements (20 or 40) have a greater range than those determined using more data points, with a tail to greater volumes particularly prominent. When more data points are added, estimated volumes become more consistent, with fewer outliers in volume (Fig. 11). A systematic decrease in volume standard deviation as dataset size increases indicates a better-constrained surface.

Volume estimates produced using datasets of less than 40 measurements results in large volume error, shown by the range in volume estimates in Fig. 11. The Askja Layer D, Taupo Plinian Pumice, and Fogo member A examples show that volume uncertainty changes little whether 80 or 100 measurements are used. Results from all simulations indicate that estimated volume decreases as more data are used; however, this is dependent on the spacing and distribution of the data points, with lower standard deviation for increased dataset size also likely related to better spread of data around the source. Based on these results, uncertainty in volume estimates associated with datasets of different sizes is estimated (Table 4). Percentage error is calculated as one standard deviation expressed as a percentage of the mean volume. These estimates also reflect error associated with the spatial distribution of measurements around the vent, with increasing dataset size leading to better representation of the deposit.

**Table 4:** Mean and standard deviation of volumes calculated using 50 subsets of ‘n’ data from each dataset

Deposit (volume)	Number of points	Median volume (km^3^)	Standard deviation (km^3^)	Volume error (%)
Fogo member A (1.0 km^3^)	20	1.73	1.00	58
	40	1.43	0.73	51
	60	1.53	0.58	37
	80	1.55	0.77	45
	100	1.28	0.54	42
Cerro Negro 1995 (0.0045 km^3^)	20	0.014	0.0072	51
	40	0.010	0.0049	49
	60	0.018	0.0089	49
Askja Layer D (0.19 km^3^)	20	0.22	0.28	127
	40	0.19	0.29	152
	60	0.15	0.09	60
	80	0.29	0.11	57
Taupo Plinian Pumice (6.7 km^3^)	20	14.60	16.50	113
	40	10.69	3.82	36
	60	12.00	3.44	28
	80	11.07	2.26	20
	100	11.83	3.29	27

Volume error was calculated as a percentage of one standard deviation of the median volume. Volume determined using spline interpolation of complete dataset in parentheses

In most cases, there is a systematic decrease in volume error with an increase in the number of measurements within each dataset. However, volume errors differ depending on deposit shape, with examples of long thin deposits (Askja Layer D), showing greater volume errors than circular to sub-circular deposits (Taupo Plinian Pumice and Fogo member A). To generalize the results displayed in Table 4, isopach maps based on N > 40 are likely to have volume uncertainties related to contouring of thickness data of between factors of 1.5 and 2. These values are consistent with those of Bonadonna and Costa (2012), who report a factor of 2 on volume uncertainties when the deposit is not fully described.

## Implications from application of the cubic B-spline method

Accurate estimation of volumes from tephra fall deposits is a major objective in many volcanological studies, and estimates have thus far predominantly been based on isopach maps interpolated from thickness measurements. Hand-drawn isopach maps rely on judgement to some extent, informed by the data, and have been shown to be highly variable dependent on the scientist (Klawonn et al. 2014a, b), with the accuracy of proximal contours being of particular importance.

Uncertainties in volume estimates are not commonly estimated and, if they are, the methodology for determination of these uncertainties is not reported. Here, we have applied a mathematical method to contour thickness data using cubic B-splines in tension. Intrinsically, it is less subjective than drawing contours by eye and enables selection of alternative best fits to data by controlled tuning of the roughness parameter. The method provides a systematic approach to comparing the performance of different empirical thinning laws and the cross-comparison of volume estimates from different datasets. It also enables characterization of important aspects of uncertainty of volume estimates by exploring how the number of thickness measurements and thickness uncertainty itself affect volume estimates. A key benefit of this method is the ability to incorporate uncertainty for each measurement. With an increased emphasis on how these uncertainties propagate to, for example, volume estimation, this method serves as a tool whereby the effect of uncertainties on individual measurements on volume estimation can be calculated and allows uncertain data (for example, measurements from incomplete sections providing information on the presence of a deposit but poor data regarding actual thickness) to be included in deposit interpretation.

An advantage to the method is that it enables, depending on input parameters chosen, identification of local patterns in deposit thickness trends where sufficiently-detailed measurements are present. However, extrapolation to locations where measurements are sparse or not present should be made with caution, especially for deposits with highly irregular depositional extents. Thus, volume estimates produced by integrating the spline-surface, and also the application of the exponential method, are likely to be minimum estimates of eruptive volume. The method is not limited to the use of thickness measurements, but may also be applied to tephra grain size data, to investigate uncertainties in isopleth maps, and in combination with the method of Carey and Sparks (1986), produce better estimates of eruption column height.

The spline-generated isopach maps are broadly similar to isopach maps drawn by highly experienced volcanologists. It is observed that in some of the examples, particularly that of the Mount St. Helens eruption, the isopachs produced here are more comparable to those determined using numerical models (e.g., Folch et al. 2010), than hand-drawn maps. This reflects the ability of the method to identify more complex depositional patterns, associated with aggregation for example, which may not be identified in the visual process of hand-drawing isopach maps.

In the cases we have studied, differences in volume are within uncertainties of the volume estimates (Tables 3 and 4). Areas enclosed by contours vary from those published and seem to be related to the shape, particularly aspect ratio of a deposit. The maximum thicknesses estimated for all examples are systematically smaller than those reported in the published record. This highlights that very proximal contours, representing the very thickest deposits and described by a relatively small number of points, are emphasised in the production of hand-drawn isopachs, despite these thickness being liable to large uncertainties due to nonlinear processes of near source dispersal.

In the study presented here, volume error associated with the number of measurements is investigated (Table 4); however, these values are likely to also be a function of sample distribution (e.g., Bonadonna and Houghton 2005; Bonadonna et al. 2015). There is a systematic decrease in volume uncertainty with bigger datasets but volume estimates tend to stabilize to approximately constant for 60 or more thickness measurements. In random sampling of data point subsets, proximal samples are often under-represented showing that volume estimates are influenced by spatial data distribution. The volume uncertainties derived here (factors of 1.5 to 2 for isopach maps based on good datasets) are significantly greater than those estimated by Engwell et al. (2013) derived from thickness measurement uncertainty alone (typically a few percent). Volume uncertainties are related to assumptions required when producing isopachs, either by hand or using a mathematical model, and the choice of integration technique. Data spacing also plays a role, with well-distributed measurements resulting in better-constrained isopachs and more reliable volume estimates. Bonadonna and Houghton (2005) highlighted the importance of sample distribution relative to source when estimating total grain size distribution of deposits and erupted volume. Proper quantification of the impact of measurement distribution on volume estimation is complex, requiring further study by application of statistical methods. It is likely that the number of contours also has a large impact on volume uncertainty. In cases where distal thickness trends are poorly defined, and therefore isopachs cannot be defined, distal trends may be overwritten by proximal trends (e.g., Bonadonna and Costa 2012 and Klawonn et al. 2014a). This factor is likely eruption type dependent, with very large eruptions whose distal deposits cannot be well defined using isopachs (e.g., the Campanian Ignimbrite eruption; Cornell et al. 1983), more liable to large uncertainties and specifically volume underestimation.

Another significant source of uncertainty is only addressed to a limited extent here. Volume estimates often require extrapolation beyond areas where there are data, and the choice of fitting function for log thickness versus isopach area is crucial. Herein, volumes are described assuming exponential decay, and thickness decay in distal locations beyond the area containing measurements is not taken into account. The examples are well described by two or fewer exponential segments, indicating the complete deposit, and particularly the distal deposit, is not well described by the measurements (Bonadonna and Houghton 2005). A number of the examples can be represented by segmented exponential decay (Fierstein and Nathenson 1992) or fit to concave power laws or Weibull functions (Bonadonna and Houghton 2005; Bonadonna and Costa 2012). These more complex functions suggest that volumes based on proximal data that display exponential thinning will be underestimated. These results reflect those from a number of theoretical and numerical studies (Sparks et al. 1992; Bursik et al. 1992; Bonadonna et al. 1998), which show the importance of sedimentation processes, particularly the role of Reynolds number on particle terminal velocity (Bonadonna et al. 1998), resulting in varying rates of thickness decay with distance from source. While these effects have been studied in proximal, medial distances, a better understanding of this source of uncertainty is likely to come from a combination of modeling studies and better empirical datasets that extend to great distances.

The application of a specific algorithm to thickness datasets would put future studies of tephra distribution on a more robust footing. However, there are some limitations and issues. Spline analysis requires large datasets to generate closed contours, and constrained extrapolation to zero is not possible in the absence of zero-valued data points. This reflects difficulties in the process of volume estimation and deposit description, whereby the full extent of a deposit is very rarely identified. An approximation of the deposit extent by identification of locations where no tephra is found to define a zero iso-line in the same manner as used in the estimation of total grain-size distribution (Bonadonna and Houghton 2005) would make tephra thickness datasets more robust, enabling detailed analysis of volume error and more accurate volume estimation. The volume uncertainties characterized here are only the component of uncertainty related to the areas with data.

Finally, this study indicates that greater attention should be paid to standardization of field measurement methods (Engwell et al. 2013), data analysis approaches, and uncertainty assessment. We believe it would materially improve the quality and comparability of isopach maps and volume estimates if defined procedures and an accepted algorithm are applied by the community. The cubic B-spline in tension is suggested as a viable option. One way in which the method could be used is to test the quality of hand-drawn maps. Our study has noted marked departures in volume and thinning rates between hand-drawn and spline generated isopach maps for deposits with good datasets and produced by experienced volcanologists. In future studies, hand-drawn maps could be adjusted to agree with spline-derived maps and comparisons could help improve skills in hand-drawing maps. We also recommend that thickness studies should include assessment of uncertainty in thickness measurements and in enumeration of volume uncertainties, with application of the spline methodology providing an objective way of doing this.

## Conclusions

We present a method for producing isopach maps objectively by applying a mathematical fitting model to tephra thickness data. Subjective information, for example, expected shape or smoothness of an isopach is not taken into account, allowing more detailed, non-uniform thickness decay patterns to be presented. The resultant isopach maps indicate tephra thickness distribution is often more complex than usually represented using simple spherical or oblate isopachs. This is partly related to localised syn- and post-depositional processes unrelated to eruption source dynamics, but also due to spatial heterogeneities in data, with data clustering and areas with sparse or no data contributing to increase uncertainty related to constructing isopach maps. Ignoring these intricacies in the data leads to inaccurate volume estimation. Volumes determined here differ by up to 40 % from those determined using published hand-drawn isopachs, mainly associated with differences in distal thinning trends. Direct integration of the produced surface to provide estimates of volume within each isopach contour interval highlights the dominance of volume contained within proximal deposits on volume estimates, consistent with the results of Klawonn et al. (2014a, b).

Uncertainties in isopach positioning are highly dependent on the number of data points. The examples studied here display large uncertainties associated with small (<60 measurements) datasets, while uncertainty varies little with datasets of larger than 60 measurements. However, this figure is likely to vary for different deposits depending on the spatial distribution of measurements, and the complexity of the deposit, for example, more measurements would be required for description of bilobate deposits. Volume uncertainties are greater than those associated with measurement uncertainty and highlight the need to detail methods used, and quantify uncertainty associated with volume estimation. Results also highlight a requirement for better appraisal of the deposit extent, with observations of locations where the deposit is absent as important as those defining substantial thickness.

## Electronic supplementary material

ESM 1 (DOCX 73 kb)

Figure A1 The effect of varying spline tension (tau = 0; 0.25; 0.5; 1.0) on the resulting Fogo member A isopach contour map. In each case, the spline roughness is set to 1.0 and knot spacing is 5 km. (PDF 713 kb)

Figure A2 The effect of spline knot spacing (20; 10; 5; and 3.5 km) on the resulting Fogo member A isopach contour map; spline tension is set to 0.99 and roughness to 1.0. (PDF 641 kb)
