# Supplementary material for: An objective method for the production of isopach maps and implications for the estimation of tephra deposit volumes and their uncertainties
Source: Bull Volcanol. 2015 Jun 17;77(7):61. doi: 10.1007/s00445-015-0942-y (PMC4498447; doi:10.1007/s00445-015-0942-y)
Supplement: Supplementary file 1 — (DOCX 73 kb) [file 445_2015_942_MOESM1_ESM.docx]

Appendix 1. Tension

A value of 0.99 was chosen for tension based on initial studies, and the results of Inoue (1986) and Bauer et al. (1998). Figure A1 shows the effect of varying tension on the resulting isopach maps. When there is no tension the derived surface is not physically feasible. When the tension is increased, the isopachs become more realistic. There is little variation in deposit thinning trends between tensions of 0.5 and 1.0 however, with error between estimated thickness and measured thickness varying by less than 1% between a tension of 0.25 (10.6 % error) and 1.0 (11.1 % error).

Appendix 2. Knot spacing

Studies to determine the effect of varying the knot spacing over the deposit extent show that results are robust within a large range of values (Figure A2). It is shown that with very low knot spacing (20 km), the surface is too smooth to accurately represent local-scale trends in the data. This is especially apparent very close to source, where the thickest deposits are poorly described. As the knot spacing is decreased, such that there are more knots over the area, the isopachs become more irregular, but there is little change in the area within each contour. Reducing the knot spacing from 20 km to 3.5 km results in errors in estimated thickness being reduced from 12% to 9%, respectively.
