# Supplementary figures and images for: An objective method for the production of isopach maps and implications for the estimation of tephra deposit volumes and their uncertainties
Source: Bull Volcanol. 2015 Jun 17;77(7):61. doi: 10.1007/s00445-015-0942-y (PMC4498447; doi:10.1007/s00445-015-0942-y)

UTM Y (m)

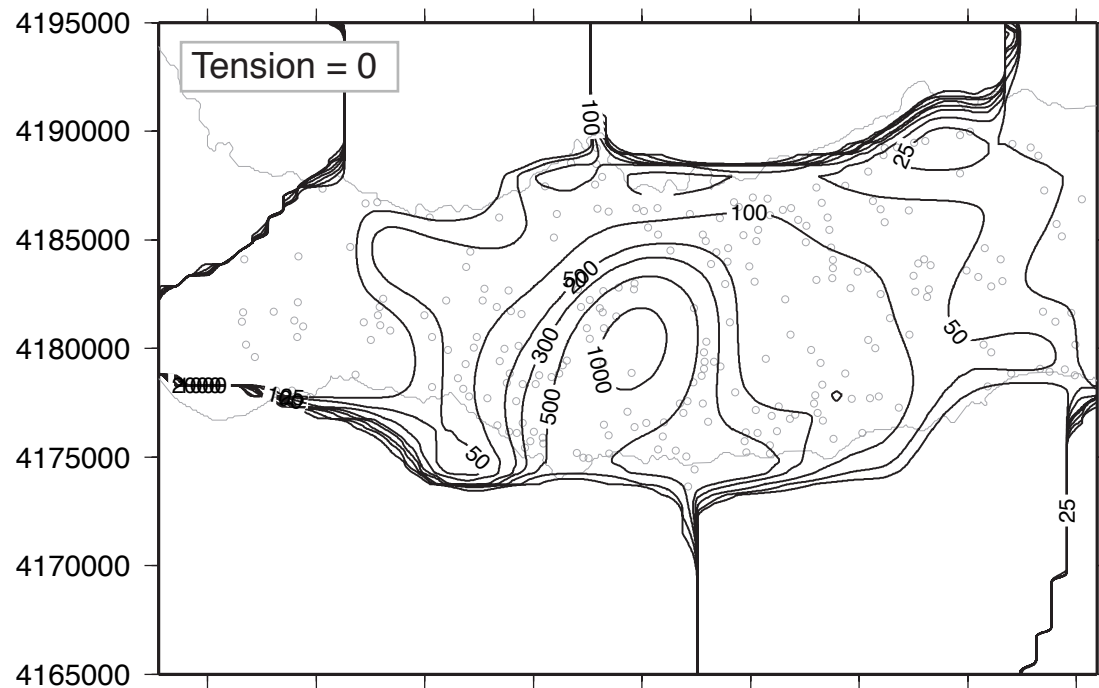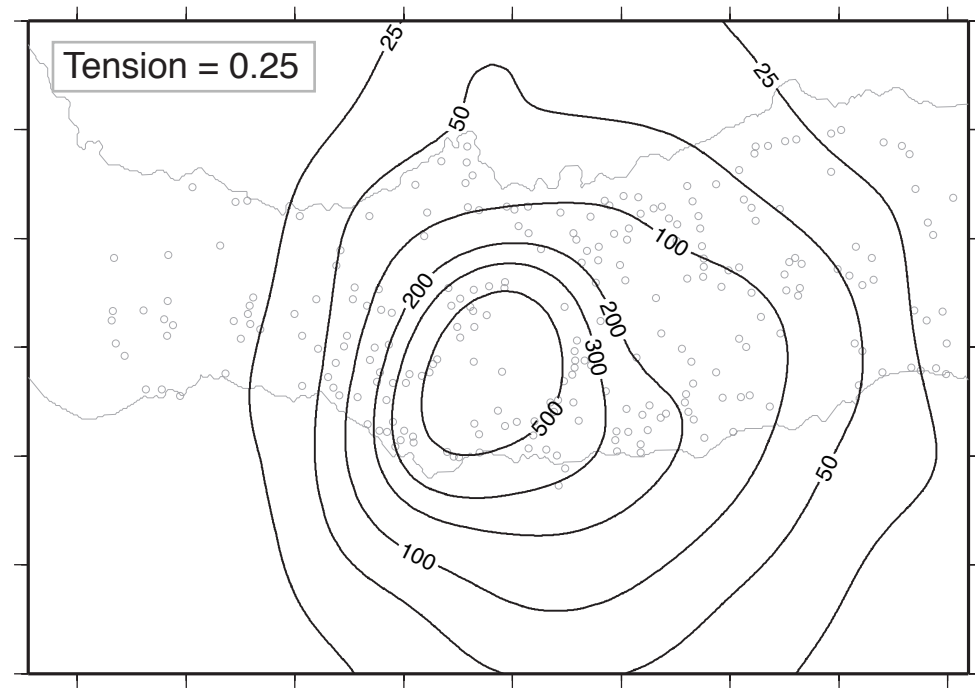

UTM Y (m)

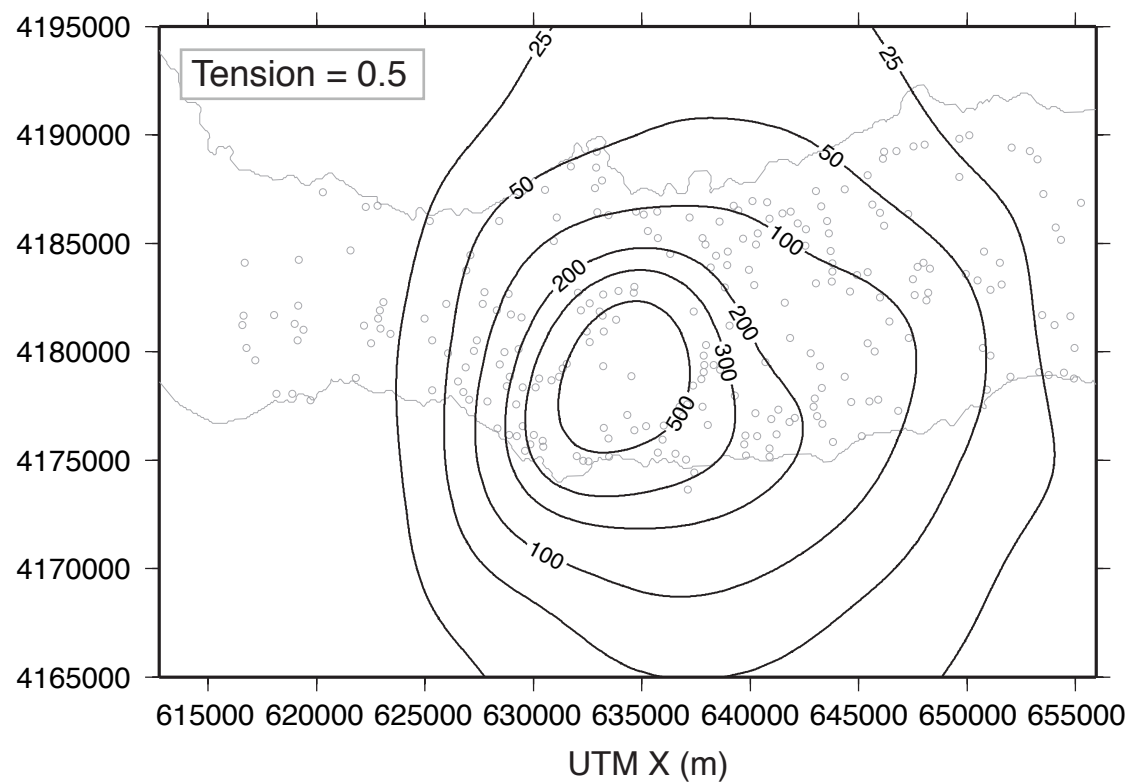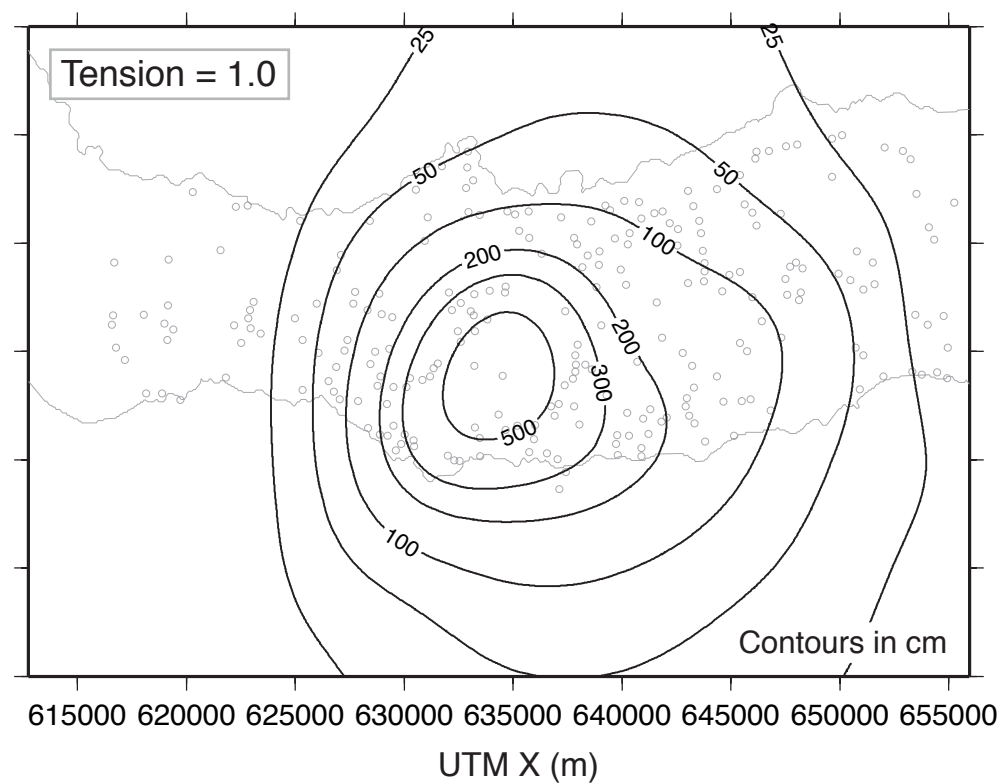

Supplement: Supplementary file 2 — The effect of varying spline tension (tau = 0; 0.25; 0.5; 1.0) on the resulting Fogo member A isopach contour map. In each case, the spline roughness is set to 1.0 and knot spacing is 5 km. (PDF 713 kb) [file 445_2015_942_MOESM2_ESM.pdf]

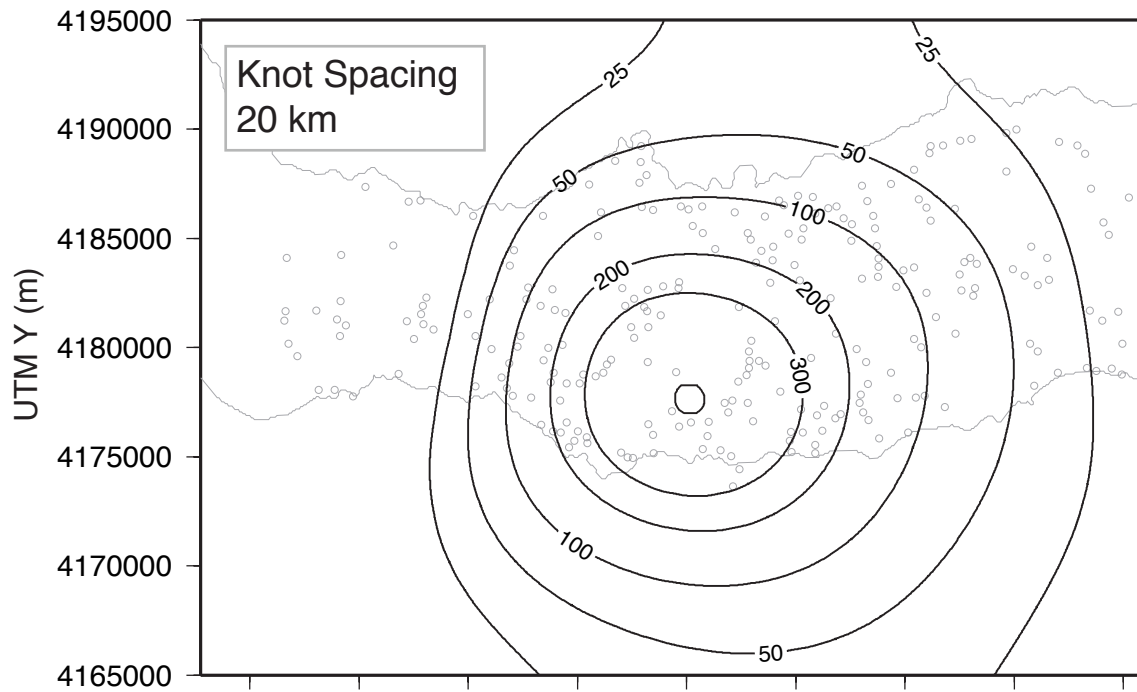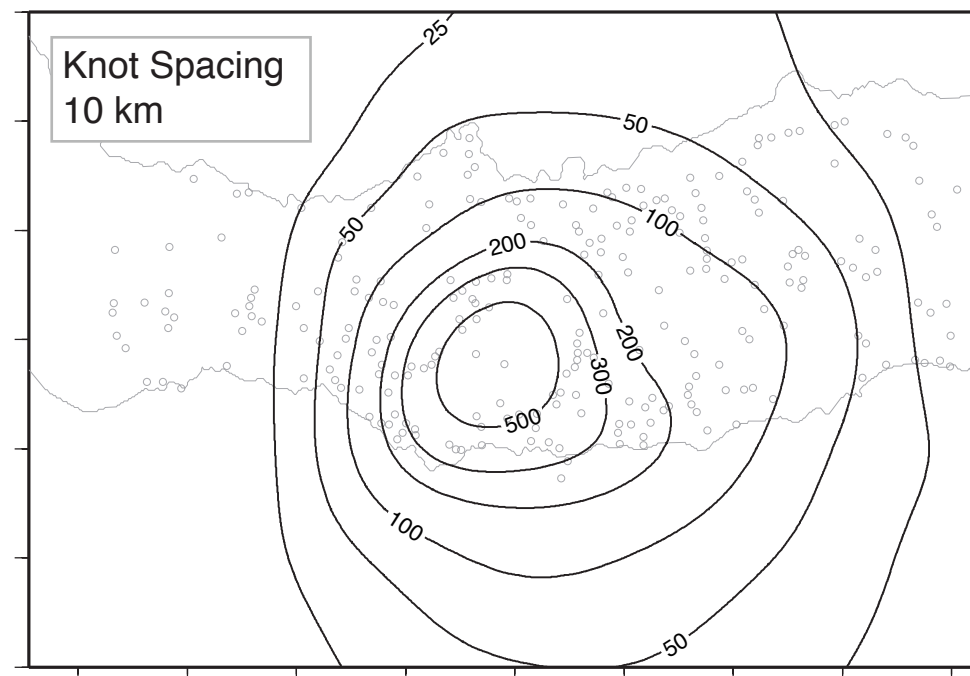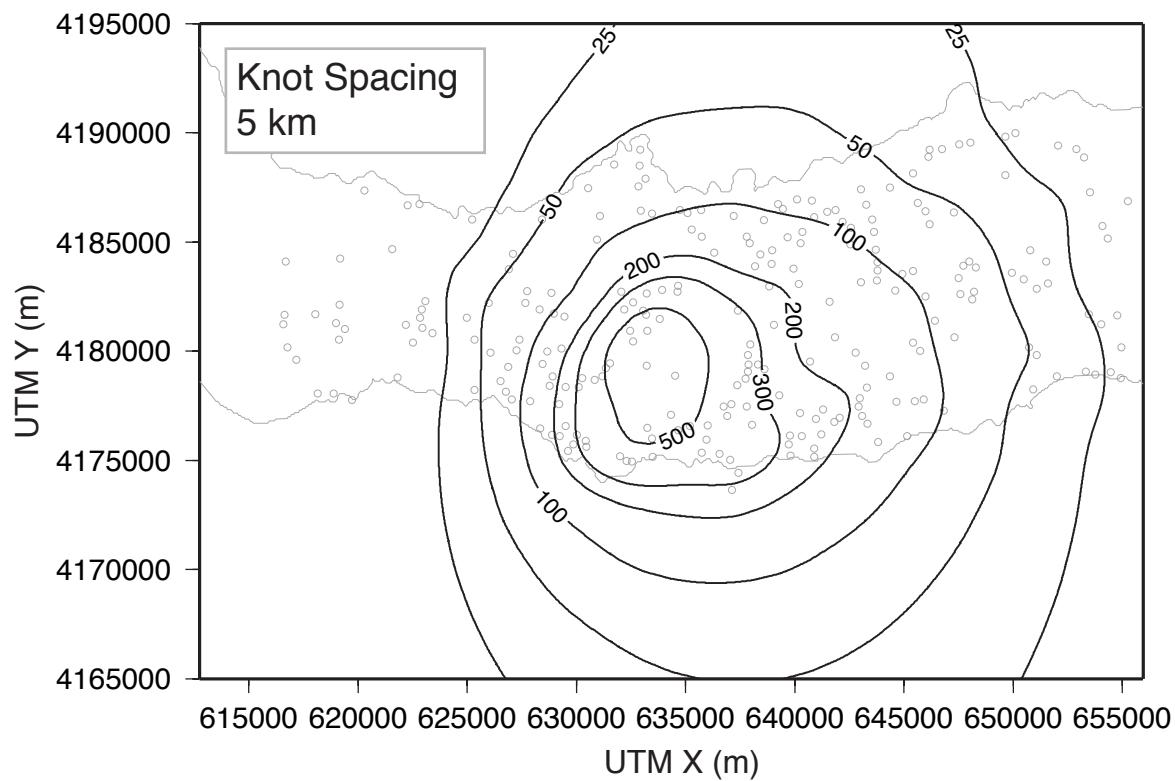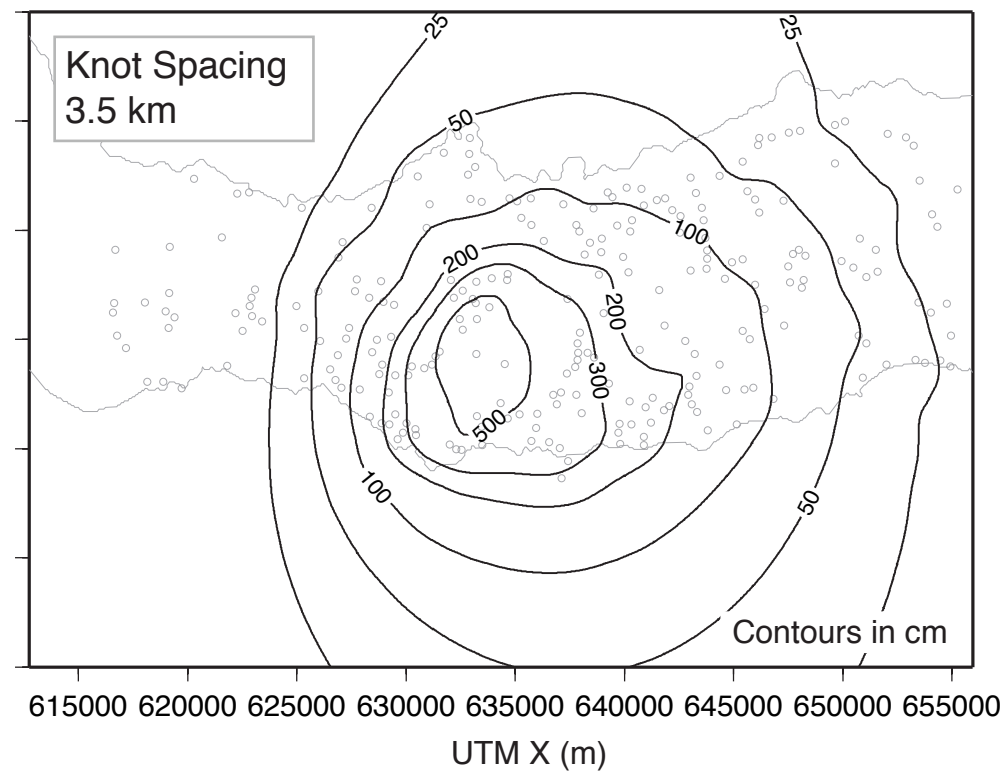

Supplement: Supplementary file 3 — The effect of spline knot spacing (20; 10; 5; and 3.5 km) on the resulting Fogo member A isopach contour map; spline tension is set to 0.99 and roughness to 1.0. (PDF 641 kb) [file 445_2015_942_MOESM3_ESM.pdf]
